# The Morphology and Solute Segregation of Dendrite Growth in Ti-4.5% Al Alloy: A Phase-Field Study

**DOI:** 10.3390/ma14237257

**Published:** 2021-11-27

**Authors:** Yongmei Zhang, Xiaona Wang, Shuai Yang, Weipeng Chen, Hua Hou

**Affiliations:** 1 School of Science, North University of China, Taiyuan 030051, China; yongmeizhang@nuc.edu.cn (Y.Z.); zbdxwxn@163.com (X.W.); 2 School of Materials Science and Engineering, North University of China, Taiyuan 030051, China; zbdx_ys@163.com (S.Y.); zbdxcwp@163.com (W.C.); 3 School of Materials Science and Engineering, Taiyuan University of Science and Technology, Taiyuan 030024, China

**Keywords:** Ti-4.5% Al alloy, phase-field method, dendrite, solute segregation

## Abstract

Ti-Al alloys have excellent high-temperature performance and are often used in the manufacture of high-pressure compressors and low-pressure turbine blades for military aircraft engines. However, solute segregation is easy to occur in the solidification process of Ti-Al alloys, which will affect their properties. In this study, we used the quantitative phase-field model developed by Karma to study the equiaxed dendrite growth of Ti-4.5% Al alloy. The effects of supersaturation, undercooling and thermal disturbance on the dendrite morphology and solute segregation were studied. The results showed that the increase of supersaturation and undercooling will promote the growth of secondary dendrite arms and aggravate the solute segregation. When the undercooling is large, the solute in the root of the primary dendrite arms is seriously enriched, and when the supersaturation is large, the time for the dendrite tips to reach a steady-state will be shortened. The thermal disturbance mainly affects the morphology and distribution of the secondary dendrite arms but has almost no effect on the steady-state of the primary dendrite tips. This is helpful to understand the cause of solute segregation in Ti-Al alloy theoretically.

## 1. Introduction

Dendrite is a common microstructure in the solidification process of pure metals and alloys. The characteristics of dendrite groswth are the non-linear evolution of the growth of the primary dendrite arm, secondary dendrite arm, and tertiary dendrite arm tips on the micro-scale. Ti-Al alloys have been widely used in the aviation and automobile industry due to their low density, good stiffness, high-temperature resistance, and good creep properties [1,2]. In the solidification process of Ti-Al alloys, solute segregation is inevitable [3,4], which will lead to the formation of the second phase and cracks [5]. Therefore, understanding the dendrite morphology and the cause of solute segregation is helpful to improve the mechanical properties of the alloys.

A great deal of research has been carried out on the factors affecting dendrite growth. The preferred orientation of dendrite [6,7] will change the dendrite morphology and dendrite arm spacing, the interface [8] and thermal disturbance [9,10] affect the morphology of secondary dendrite arms, and the competitive growth [11] and convection [12,13,14] affect the direction of dendrite growth. Karma and Rappel [15] broadened the width of the diffusion interface and simulated the dendrite evolution of pure metals in two-dimensional (2D) and three-dimensional (3D) [16]. Ohno and Matsuura [17,18] extended the phase-field model developed by Karma to contain the solid diffusion coefficient. Liu et al. [19] simulated the dendrite growth process of Ti-6Al-4V alloy and revealed the details of the formation of the secondary dendrite arms. Sun et al. [20] used the multi-component phase-field model to conduct 2D and 3D simulations of Ti-6Al-4V alloy and found that the phenomenon of dendrite merger will increase the dendrite spacing and coarse dendrite. Feng et al. [21] simulated the growth process of the equiaxed dendrite of Al-Cu alloy based on the multi-component phase-field model, and the simulation results showed that the dendrite competitive growth of multiple grains. Chen et al. [22] studied the growth process of the equiaxed dendrite of Al-Cu alloy based on a 2D phase-field model and performed synchronous X-ray in-situ characterization. The results showed that when dendrite grows freely and is far away from neighboring dendrite, the tip growth rate of the dendrite will increase with the increase of the undercooling, and when two equiaxed dendrites interact, the tip speed will reach the maximum when the dendrite arms are in contact with each other, and then gradually decrease to zero. Zhao et al. [23] studied the effect of thermal coupling strength on the growth of pure Ni dendrite, and the results showed that the thermal coupling strength will affect the front of the dendrite interface, leading to secondary dendrite arms coarsening. Zhang et al. [24] used the phase-field model developed by Karma to simulate the influence of the interface width and thermal disturbance on the equiaxed dendrite growth of Al-Si alloy. The results showed that the increase of the interface width will cause the growth rate of the dendrite tip to decrease, and the thermal disturbance will affect the number and morphology of secondary dendrite arms. Boukellal et al. [25] conducted a 3D phase-field simulation of Al-Cu equiaxed dendrite growth. The simulation results showed that the existence of thermal fluctuations is essential at lower concentrations, and thermal fluctuations will affect the development of secondary dendrite arms. Zhang et al. [26] simulated the solute segregation of Ti-45Al alloy in liquid→liquid+β(Ti) via phase-field model combined with thermodynamics calculations, and the results showed that with the growth of β(Ti) phase, dendritic segregation is formed in the liquid and solid phases. As the solidification progresses, the growth rate of the dendrite tip decreases, and the segregation rate increases. Li et al. [27] simulated the growth process of the equiaxed dendrite of Ti-Al alloy under isothermal and non-isothermal conditions, and the results showed that the initial composition and temperature have a great influence on the dendrite morphology and solute distribution, which will cause the dendrite structure to be underdeveloped, and the lower interface composition. Viardin et al. [28] used an envelope phase-field model to simulate the growth of equiaxed dendrite in Ti-45at.% Al alloy under forced convection, and the results showed that the growth of equiaxed dendrite is asymmetric.

Many studies [29,30,31] have proved the feasibility of the phase-field model developed by Karma [32,33] for quantitative simulation. For instance, Becker et al. [29] combined X-ray in situ imaging, EBSD analysis, and phase-field modeling to study the dendrite orientation transition in Al-Ge alloy. Dantzig et al. [30] observed a transition of dendrite orientation as a function of the Zn content in Al-Zn alloy. Hing et al. [31] studied the orientation dependence of the columnar dendritic growth with side branching behaviors in directional solidification of a binary alloy and found that the dimensionless tip undercooling in direction solidification is an increasing function of the misorientation angle. Moreover, the phase-field method is closely related to thermodynamic as proposed definitely by Chen and Zhao in [34], and the real thermodynamic parameters of the material can be related to the parameters of the phase-field model one by one. For instance, based on the thermodynamic parameters of magnesium-based alloy and the phase-field method, Xin and Zhao et al. [35] successfully revealed the basic principle of spinodal decomposition strengthening magnesium alloy.

In this work, based on our previous studies [36,37,38,39,40,41,42], the morphology and solute segregation of solidification dendrite growth of Ti-4.5% Al alloy is numerically simulated by using the phase-field model with the thin interface approximation proposed by Karma. As the model is suitable for dilute solution, the Ti-4.5% Al alloy system is selected to ensure the accuracy of the simulation. The effects of supersaturation, undercooling, and thermal disturbance on dendrite growth morphology are studied, and some reasons for solute segregation are obtained, which is helpful to understand the cause of solute segregation in Ti-Al alloy theoretically.

## 2. Phase-Field Model and Simulation Parameters

### 2.1. Phase-Field Model

Here, the improved binary alloy phase-field model developed by Ohno and Matsuura is used [18]. It includes phase-field governing equation, solute-field governing equation, and temperature-field governing equation:

(1) $$\tau \frac{\partial \phi }{\partial t}=W^{2}\nabla^{2}\phi +\left(\phi -\phi^{3}\right)-\frac{\lambda }{1-k_{e}}{\left(1-\phi^{2}\right)}^{2}\left(e^{u}-1\right)$$

(2) $$\frac{\partial c}{\partial t}=\nabla \cdot \left[D_{l}e^{u}\frac{c_{l}^{0}\left[1-\phi +k_{e}\frac{D_{s}}{D_{l}}\left(1+\phi \right)\right]}{2}\nabla u\right]-\nabla \cdot j_{at}$$

(3) $$\frac{\partial \theta }{\partial t}=D_{T}\nabla^{2}\theta +\frac{1}{2}\frac{\partial \phi }{\partial t}-\nabla \cdot q\left(\vec{r},t\right)$$

In the equations above, the dimensionless parameter *u* representing the solute concentration is given by

(4) $$u=ln\frac{c}{\left[\left(c_{l}^{0}+c_{s}^{0}\right)/2+\phi \left(c_{l}^{0}+c_{s}^{0}\right)/2\right]}$$

where $c_{s}^{0}$, $c_{l}^{0}$ is the equilibrium solute concentration of liquid and solid phases, $c_{s}^{0}=k_{e}c_{l}^{0}$, τ is the relaxation time, W is the width of the interface, and λ is the dimensionless phase-field parameter, $\lambda =a_{1}W/d_{0}$, where $a_{1}$ is 0.8839, $d_{0}$ is the solute capillary length, $d_{0}=-\Gamma_{sl}/\left[m\left(1-k_{e}\right)c_{l}^{0}\right]$, m is the slope of the alloy liquidus, $k_{e}$ is the solute equilibrium distribution coefficient, $D_{s}$, $D_{l}$ are the solid/liquid solute diffusion coefficients, $j_{at}$ is the anti-solute interception term, and θ is the dimensionless temperature, $\theta =\left(T-T_{m}\right)/\left(L/c_{p}\right)$, $T_{m}$ is the melting point temperature, L is the latent heat, $c_{p}$ is the constant volume heat capacity of the alloy, $D_{T}$ is the thermal diffusion coefficient, and $\nabla \cdot q\left(\vec{r},t\right)$ is the thermal disturbance term.

When simulating the disturbance at the interface in the process of dendrite growth, the thermal disturbance needs to be considered in the phase-field model. This work introduces a random variable matrix $q\left(\vec{r},t\right)$ related to space and time in the control equations of the solute-field and temperature-field. Its average value is zero and has a Gaussian distribution, and its function expression is [43]:

(5) $$\langle q_{m}\left(\vec{r},t\right),q_{n}(\vec{r},t)\rangle =2DF_{u}\delta_{mn}\left(\vec{r}-\vec{r^{\prime }}\right)\delta \left(t-t\prime \right)$$

(6) $$F_{u}=\frac{k_{B}T_{m}^{2}c_{p}}{L^{2}W_{0}^{2}}=\frac{k_{B}T_{m}^{2}c_{p}}{L^{2}d_{0}^{2}}{\left(\frac{d_{0}}{W_{0}}\right)}^{2}=F_{excp}{\left(\frac{d_{0}}{W_{0}}\right)}^{2}$$

where $k_{B}$ is Boltzmann constant, $F_{u}$ is thermal disturbance amplitude.

During the solidification of alloys, the solid/liquid interface can possess anisotropy. In general, the preferred orientation of dendrite will generally be the direction with the smallest interface energy. For cubic symmetric crystals, the expression of the interface energy anisotropy $\;A\left(\vec{n}\right)$ is

(7) $$A\left(\vec{n}\right)=A_{0}[1+a_{1}(Q-\frac{3}{5})+a_{2}(3Q+66S-\frac{17}{7})]$$

where $A_{0}$ is the average interface energy, $\vec{n}$ is the interface normal vector, $a_{1}$ and $a_{2}$ are the anisotropic strength, Q and S are the third harmonic form, $Q=n_{x}^{4}+n_{y}^{4}+n_{z}^{4}$, $S=n_{x}^{2}n_{y}^{2}n_{z}^{2}$. The interface energy anisotropy is introduced to the phase-field equations by $W=W_{0}A\left(\vec{n}\right)$ and $\tau =\tau_{0}A^{2}\left(\vec{n}\right)$, where $W_{0}$ and $\tau_{0}$ are the spatial scale and time scale, respectively.

### 2.2. Initial Conditions and Boundary Conditions

In this work, the initial nucleus of the dendrite is set in the corner of the simulation region, and the radius is $r_{0}=10W_{0}$. Using the spherical initial interface and Zero-Neuman boundary conditions, the volume of the 3D calculation region reaches ${\left(384W_{0}\right)}^{3}$, the initial conditions are as follows:

(8) $$\{\begin{array}{c} x^{2}+y^{2}+z^{2}\leq r_{0}^{2},\;\;\phi =1\\ x^{2}+y^{2}+z^{2}>r_{0}^{2},\;\;\phi =-1 \end{array}$$

### 2.3. Other Parameters

To ensure the accuracy and convergence of the simulation results, the simulation conditions need to be met $W_{0}/d_{0}\leq 50$, which $d_{0}=5\times 10^{-9}\;m$, so the initial thickness of the interface is $W_{0}=1\times 10^{-7}m$, therefore $W_{0}/d_{0}=20$ meet the requirement. The relaxation time is related to the diffusion coefficient of the solute in the solid/liquid phase, then $\tau_{0}=2.1\times 10^{-5}\;s$. The physical parameters of the Ti-4.5% Al alloy used in this work are listed in Table 1.

## 3. Simulation Results and Discussions

### 3.1. Morphology of Equiaxed Dendrite Growth in Ti-Al Alloy

**Figure 1:**
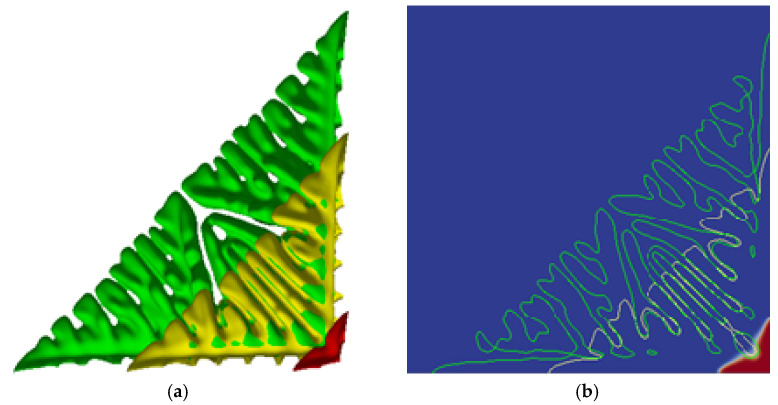
The morphology change of equiaxed dendrite growth. (**a**) Morphology of dendrite with a step size of 2000 (red), 8000 (yellow), and 12,000 (green); (**b**) Comparison of dendrite profile at the interface with a step size of 2000 (red), 8000 (yellow), and 12,000 (green).

The simulation results of the equiaxed dendrite growth process are shown in Figure 1. As shown in Figure 1a, the dendrite grows from the initial interface into a primary dendrite with a tip and valley morphology, as the solidification progresses, the number of secondary dendrite arms increases and the morphology of the dendrite becomes complicated. The secondary dendrite arms become the main growth part, and a small number of tertiary dendrite arms are produced, as shown by the yellow dendrite, then the secondary dendrite arms continue to grow and become coarser. At this time, the tertiary dendrite arms begin to grow in large quantities, and the tertiary dendrite arms become the main growth part. The dendrite necking becomes severe, and secondary dendrite arms fusion and competitive growth occur. The fusion and competitive growth of secondary dendrite arms produce coarser secondary dendrite arms, and dendrite fusion also results in the appearance of interdendritic pores, in which there will be serious solute segregation, the green irregular circle in Figure 1b is one of the pores, and there will be solute enrichment in the gaps between the secondary dendrite arms, causing solute segregation phenomenon. In the subsequent dendrite growth process, the tertiary dendrite arms will become coarse, and the phenomenon of fusion and competitive growth of the tertiary dendrite arms will appear.

### 3.2. The Effect of Supersaturation on Dendrite Morphology

**Figure 2:**
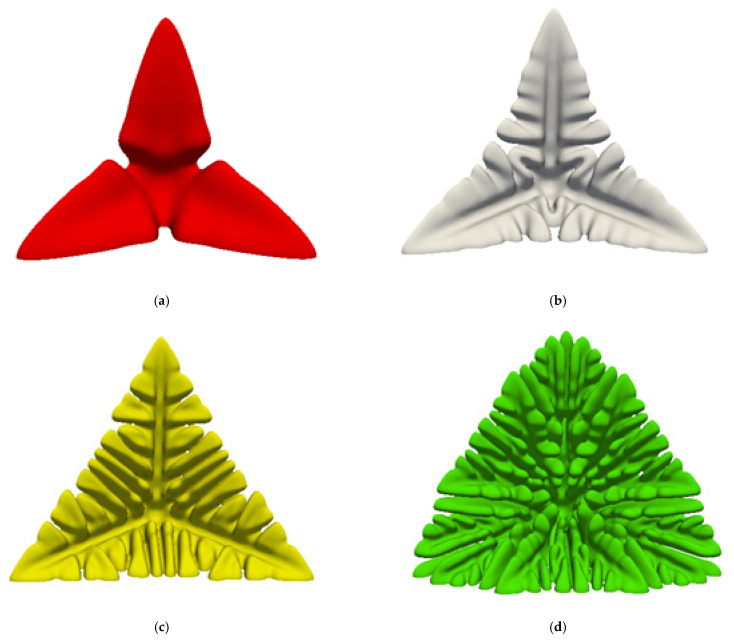
Morphology of dendrite growth under different supersaturation when the length is 8000. (**a**) Ω=0.35; (**b**) Ω=0.45; (**c**) Ω=0.50; (**d**) Ω=0.55.

Supersaturation Ω [45] is defined as the ratio of the solute change $\Delta C\left(C_{l}^{*}-C_{0}\right)$ at the tip of the dendrite to the equilibrium change $\Delta C^{*}\left(C_{l}^{*}\left[1-k\right]\right)$, it is the performance of the driving force of solute diffusion at the tip of the dendrite. The greater the supersaturation, the greater the growth rate of the newly formed phase, that is, the greater the growth rate of the dendrite, the more secondary and tertiary dendrite arms, and the solute discharge rate and growth rate are both affected by the shape of the dendrite tip, so the dendrite tip morphology will be different with different supersaturation. Figure 2 shows the morphology of dendrite growth under different supersaturations at 8000 steps. As can be seen from Figure 2a, when the supersaturation is small, the overall dendrite morphology is simple, it is a primary dendrite morphology, and there is a tendency of secondary dendrite arm growth. The primary dendrite arms have an obvious necking phenomenon, and the roots appear concave, which is easy to cause solute enrichment; when the supersaturation increases, as shown in Figure 2b, the number of secondary dendrite arms increases significantly, and there is a small amount of coarse secondary dendrite arms, the necking phenomenon of primary dendrite arms become more serious, and dendrite gaps may occur; when the supersaturation reaches 0.50, as shown in Figure 2c, there are a lot of coarse secondary dendrite arms, and there are small protrusions of tertiary dendrite arms on the secondary dendrite arms, and the necking phenomenon of the primary dendrite arms disappear, when the supersaturation reaches 0.55, as shown in Figure 2d, it can be found that the overall morphology of the dendrite is particularly coarse. The coarse secondary dendrite arms fill the entire primary dendrite arms, and a large number of tertiary dendrite arms are produced. The necking phenomenon of the primary dendrite arms disappear, and the morphology of the tips of the dendrite change. This result is consistent with the simulation results of Dantzig et al. [30].

**Figure 3:**
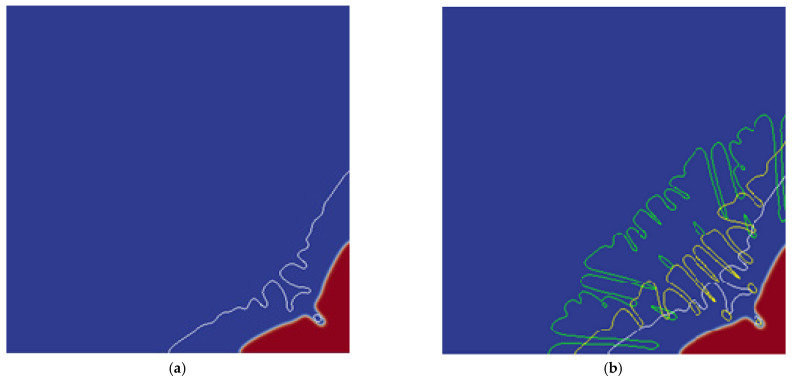
Comparison of the morphology and profile of dendrites at the interface under different supersaturations at a step size of 8000. (**a**) Comparison of supersaturation 0.35 and 0.45; (**b**) Comparison of supersaturation 0.35, 0.45, 0.50, and 0.55. Where red is 0.35, white is 0.45, yellow is 0.50, and green is 0.55.

Figure 3 shows the comparison of the morphology and profile of the dendrite at the interface with different supersaturation at 8000 steps. It can be seen from Figure 3a that the growth morphology of the dendrite with a supersaturation degree of 0.45 is larger than that of 0.35, and there are obvious secondary dendrite arms and valleys between the primary dendrite arms. It is even more obvious, that is, the solute enrichment in the valley is more serious, the solute segregation is more serious, and there is a pore between dendrites, and the pore is the most serious solute enrichment. It can be seen from Figure 3b that there are three pores in the yellow dendrite with supersaturation of 0.50, and the number of secondary dendrite arms increases; while the morphology of the green dendrite with supersaturation of 0.55 is the most complicated, the secondary dendrite arms coarse, fusion, the morphology of the tip of the dendrite changes significantly, there are 6 pores between the dendrite, and there is no obvious necking phenomenon.

**Figure 4:**
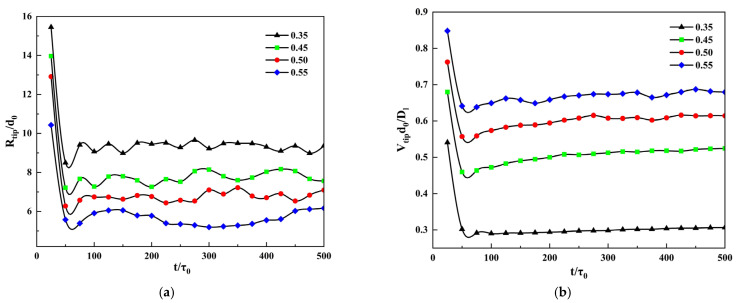

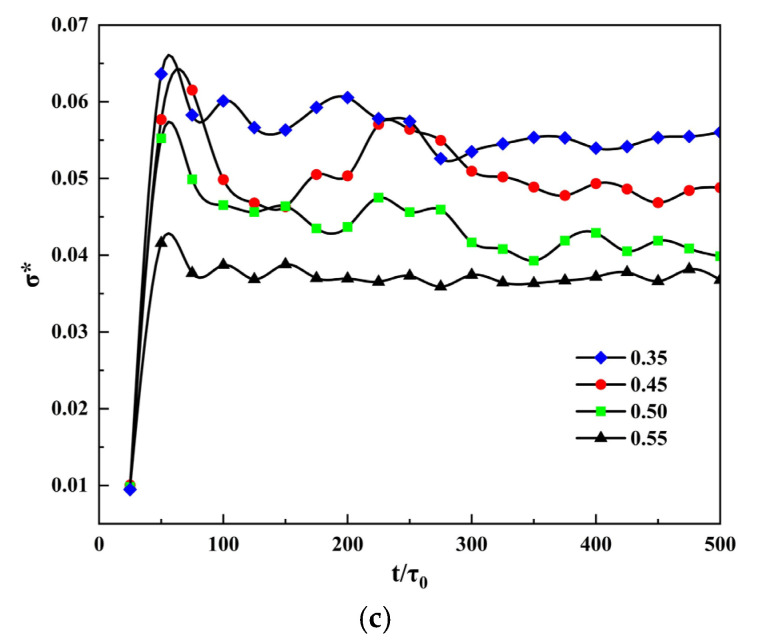
The curve of dendrite tip parameters changes with simulation time under different supersaturation. (**a**) Tip velocity; (**b**) Tip radius; (**c**) Steady-state coefficient.

Figure 4a shows the change of the dendrite tip velocity under different supersaturations, the tip velocities of the dendrite under all supersaturation are very large at the beginning because the initial nuclei easily grow in the undercooling melt. As the dendrite grows, the solute diffuses and enriches to the tip, causing the tip velocity to decrease. As the solidification time continues to advance, the solute discharge rate during the dendrite growth process and the solute diffusion rate in the liquid phase reach a balance, and the dendrite growth reaches a stable state, at this time, the dendrite tip speed also remains stable. It can be seen from the change of the dendrite tip velocity under different supersaturation that the supersaturation will directly affect the dendrite growth process. The greater the supersaturation, the greater the velocity of the dendrite tip, and the easier the solute diffusion at the tip, the speed will be greater. When the supersaturation is 0.55, the morphology of the tip of the dendrite changes in the later stage of the solidification time, and the secondary and tertiary dendrite arms become the main growth parts, resulting in a decrease in the tip velocity of the primary dendrite arms. It can be seen from the simulation results that the supersaturation affects the growth of dendrite, the greater the supersaturation, the faster the growth rate of the dendrite, the number of secondary dendrite arms increases, and the morphology is more complex.

Figure 4b,c shows the change of tip radius and steady-state coefficient under different supersaturation, respectively. The steady-state coefficient equation is [46] $\sigma^{*}=2D_{l}d_{0}/R^{2}V$, where $\sigma^{*}$ is the steady-state coefficient, $D_{l}$ is the liquid phase solute diffusion coefficient, $d_{0}$ is the solute capillary length, R is the tip radius, and V is the tip velocity. It can be seen from Figure 4b that as the solidification progresses, the tip radius of the dendrite continues to decrease and finally reaches a stable state, this is because the dendrite changes from the initial nucleus to the primary dendrite with tips and valleys, the radius of the tip will decrease accordingly. Moreover, the higher the supersaturation, the faster the growth velocity of the dendrite tip, which is opposite to the variation of the radius of the dendrite tip. It can be seen from Figure 4c that the steady-state coefficient fluctuates greatly in the early stage of dendrite growth, but in the later stage of dendrite growth, the value of the steady-state coefficient tends to be stable. This is due to, in the initial stage of dendrite growth, because the growth velocity and radius of dendrite tip are not stable, the value of $R^{2}V$ is also unstable, that is, the stability parameter $\sigma^{*}$ is constantly changing. With the progress of solidification, the dendrite growth is stable and $\sigma^{*}$ tends to be stable. With the increase of supersaturation, the time required for $\sigma^{*}$ to reach steady-state decreases. The simulation results are consistent with the Ivantsov function.

### 3.3. The Influence of Undercooling on Dendrite Morphology

**Figure 5:**
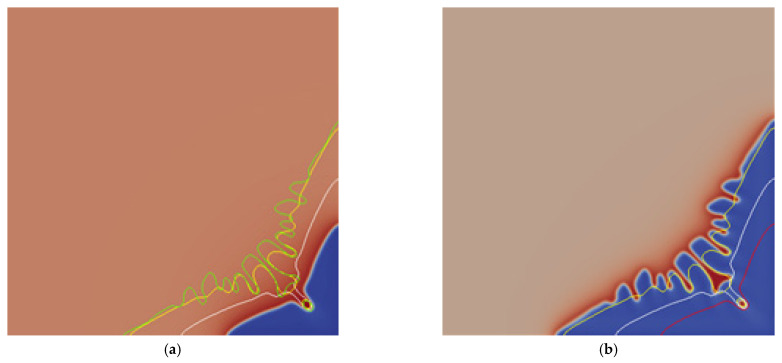
Solute-field diagram of dendrite morphology under different undercooling. (**a**) Solute-field diagram based on ΔT=20 K; (**b**) Solute-field diagram based on ΔT=35 K, where the red line, white line, and yellow line and green line are the morphological contour lines of the dendrite when the undercooling is 20 K, 25 K, 30 K, 35 K, respectively.

The undercooling affects the rate of solute diffusion, thereby affecting the growth rate of the tip and the morphology of the dendrite. Figure 5 is the solute-field diagram of the influence of different undercooling on the dendrite morphology. The red line, white line, yellow line, green line, and solid line in Figure 5a,b are the dendrite morphology contour line when the undercooling is 20 K, 25 K, 30 K, 35 K, and the base undercooling solute distribution, respectively, the solute-field diagram more intuitively reflects the solute distribution during the dendrite growth process. It can be seen that when ΔT=20 K, the dendrite morphology are primary dendrite arms with tips and valleys, the surface of the primary dendrite arms are smooth and the roots appear solute enrichment; when the undercooling increases to 25 K, the dendrite is still dominated by primary dendrite arms, and small protrusions of secondary dendrite arms appear on the surface of primary dendrite arms; after the undercooling continues to increase, the overall morphology of dendrite becomes coarse, have a large number of secondary dendrite arms, and a solute-rich hole appears at the roots. When the undercooling reaches 35 K, the secondary dendrite arms will be more developed, the number will further increase, and the morphology will become coarser. There is a solute-rich hole inside the dendrite, and the roots of the primary dendrite arms will appear very severe solute enrichment. It can be found that as the undercooling increases, the morphology of the dendrite growth becomes larger, and the solute enrichment at the root of the primary dendrite arms becomes more serious. Due to changes in the undercooling, the temperature gradient at the solid/liquid interface will change, solute diffusion rate and growth rate will also change, resulting in changes in interface stability and dendrite morphology.

### 3.4. The Influence of Thermal Disturbance on Dendrite Morphology

**Figure 6:**
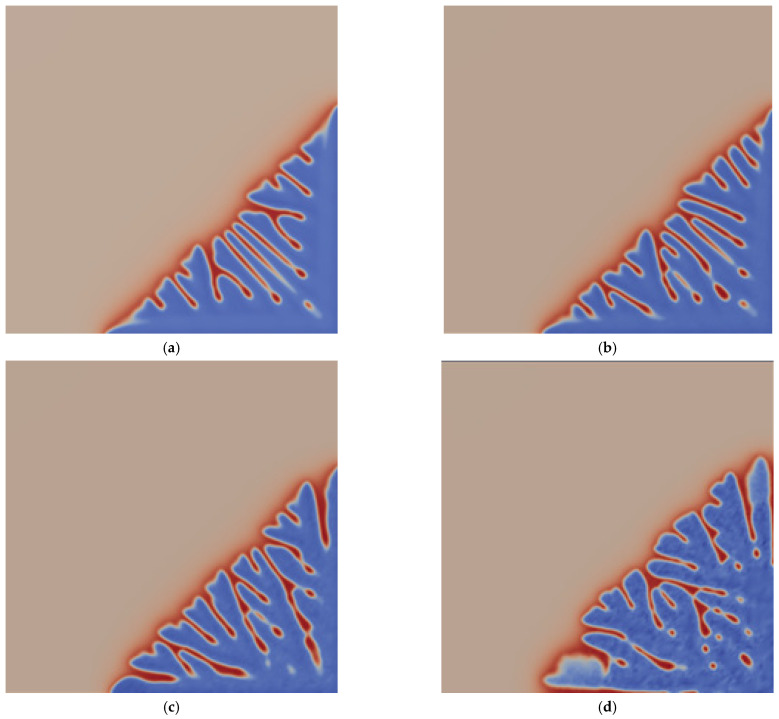
Solute-field diagram of dendrite morphology under different thermal disturbance values when the step size is 9000; (**a**) $F_{u}=0$; (**b**)$\;F_{u}=10^{-5}$; (**c**)$\;F_{u}=10^{-4}$; (**d**)$\;F_{u}=10^{-3}$.

In the process of dendrite growth, the thermal disturbance will cause the solid/liquid interface to become unstable, resulting in a more complex dendrite morphology. Figure 6 shows the solute-field diagram of dendrite morphology under different thermal disturbance values at 9000 steps. It can be seen from Figure 6 that when there is no thermal disturbance, as shown in Figure 6a, the dendrite have a symmetrical morphology, with fewer pores between the dendrite, and less solute enrichment; when the thermal disturbance increases, it can be found that the overall morphology of the dendrite is not much different, but the secondary dendrite arms become asymmetrical, the morphology becomes irregular, and how much pores are required compared to the no thermal disturbance situation, the solute segregation phenomenon is also slightly more serious; as the thermal disturbance continues to increase, as shown in Figure 6c, the overall dendrite morphology becomes more irregular, asymmetry increases, dendrite fusion phenomenon increases, and solute segregation becomes more serious; when the thermal disturbance reaches $F_{u}=10^{-3}$, the dendrite morphology has obvious asymmetry, the number of secondary and tertiary dendrite arms increases, the fusion of dendrite is more obvious, and the number of pores between dendrite is the largest, and dendrite segregation is also particularly serious. From the simulation results, it can be seen that as the thermal disturbance increases, the asymmetry of the dendrite will increase, the fusion between the dendrite will become more frequent, and the number of pores will also increase, indicating that thermal disturbance will significantly affect the growth of secondary dendrite arms, leading to the asymmetry of the dendrite, but has little effect on the overall morphology of the primary dendrite arms. This result is consistent with the simulation results of Zhu et al. [47].

**Figure 7:**
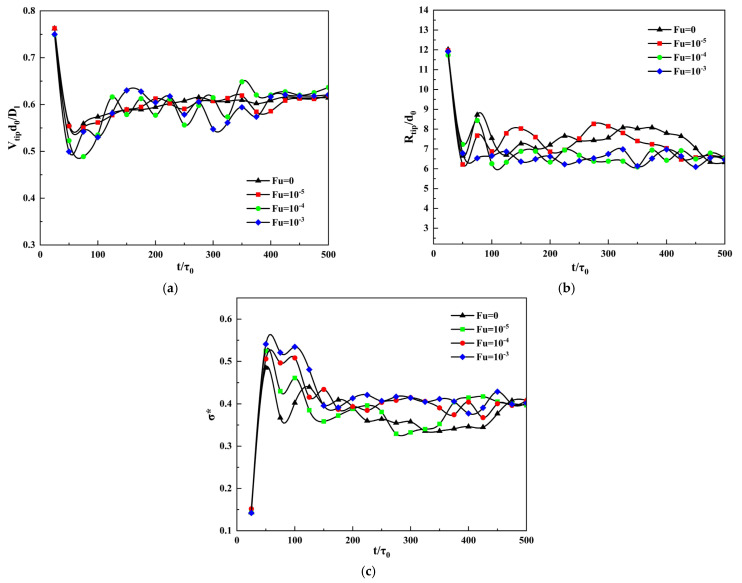
The curve of dendrite tip parameters changes with simulation time under different thermal disturbances. (**a**) Tip velocity; (**b**) Tip radius; (**c**) Steady-state coefficient.

Figure 7 shows the change curve of tip parameters with simulation time under different thermal disturbances, where a, b, and c are the changes of the tip velocity, tip radius, and steady-state coefficient, respectively. It can be seen from Figure 7a,b that as the simulation time progresses, the tip velocity and tip radius of the dendrite fluctuate in the early period, but will gradually stabilize in the later period. From Figure 7c can be seen that the steady-state coefficient gradually stabilizes in the later stage. It can be seen from Figure 7 that the dendrite tip velocity, tip radius, and steady-state coefficient will eventually tend to the same stable value as the thermal disturbance changes, this shows that thermal disturbance will not affect the steady-state of the tip of the dendrite. Although the overall variation trend of the three curves under different thermal disturbances is similar to that of different supersaturation, different from supersaturation, there is no obvious relationship between the values of the three curves and the magnitude of thermal disturbance.

In this part, the effects of supersaturation, undercooling and thermal disturbance on the dendrite morphology and solute segregation were displayed and discussed. In many studies of phase-field simulation, experimental verification is an indispensable part. However, supersaturation and thermal disturbance are model parameters, which can not be quantitatively controlled in solidification experiments, it is difficult to realize, so we will design the experimental scheme in the later research, to realize the quantitative comparison with the simulation results. This work is used to explain in detail our study in simulation.

## 4. Conclusions

In this work, the phase-field method is used to simulate the growth of equiaxed dendrite, and the effect of supersaturation, undercooling and thermal disturbance on the growth morphology and solute segregation. The following conclusions are drawn:

(1) As the solidification process progresses, the first dendrite with tips and valleys is formed, and then the secondary dendrite arms begin to grow rapidly, making the dendrite morphology more complicated and having many small protrusions. When the secondary dendrite arms become coarse, the tertiary dendrite arms form and grow rapidly, and the morphology becomes more complex. Pores are formed between the secondary dendrite arms, and solute segregation is formed.
(2) As the supersaturation increases, the growth rate of dendrite becomes faster, the morphology of secondary dendrite arms is developed, the morphology is complex, the dendrite necking disappears, and the solute segregation becomes more serious. As the supersaturation increases, the steady-state coefficient of the dendrite will decrease.
(3) As the undercooling increases, the growth rate of dendrite accelerates, and the solute concentration in the roots of dendrite during the undercooling process is serious.
(4) As the thermal disturbance increases, the influence on the morphology of the primary dendrite arms becomes smaller, and the asymmetry of the secondary dendrite arms increases significantly. When the solute segregation is serious, the thermal disturbance will not affect the steady-state of the dendrite tip.

A natural extension of this work is to verify the simulation results through relevant experiments. We will design the experimental scheme in the following work to achieve a quantitative comparison with the simulation results.

## Figures and Tables

**Table 1:** Physical properties of Ti-4.5% Al alloy [44].

Physical Parameter	Values	Unit
$T_{m}^{Al}$	933.37	K
$T_{m}^{Ti}$	1933	K
$D_{L}$	$2.8\times 10^{-6}$	${\mathrm{cm}}^{2}/s$
$D_{S}$	$3.0\times 10^{-9}$	${\mathrm{cm}}^{2}/s$
$k_{e}$	0.6561	-
ρ	3.8	$g/{\mathrm{cm}}^{3}$
L	435.4	J/g
$c_{p}$	33.732	J/mol °C

## Data Availability

All data are available within the manuscript.
